# The HUSH Project: Using codesign to reduce sleep disruptions for patients in hospital

**DOI:** 10.1111/hex.13881

**Published:** 2023-09-23

**Authors:** Corey Adams, Ramesh Walpola, Anthony Schembri, Reema Harrison

**Affiliations:** ^1^ Australian Institute of Health Innovation (AIHI) Macquarie University Sydney Australia; ^2^ School of Population Health University of New South Wales (UNSW) Sydney Australia; ^3^ School of Health Sciences University of New South Wales (UNSW) Sydney Australia; ^4^ St Vincent's Health Network Sydney Australia

**Keywords:** codesign, improvement, patient experience, quality, sleep

## Abstract

**Background:**

Poor quality sleep in hospitals may be problematic for patients, negatively impacting their recovery and wellbeing. This project aimed to investigate the effectiveness of codesign in addressing key issues affecting sleep disruption in the healthcare setting.

**Methods:**

Codesign with patients, staff and consumer representatives was conducted in an acute metropolitan tertiary public hospital in Sydney, Australia. Through a four‐stage process, a multimodal intervention to address and reduce the impact of sleep disruptions among hospital inpatients was created. Pre‐ and post‐intervention evaluation was used to determine changes in patient‐reported sleep disruption.

**Results:**

‘The HUSH Project’ (Help Us Support Healing) intervention resulted from the codesign process, which included the provision of HUSH Sleep Packs (with earplugs, eye masks and herbal tea), patient information resources, and ward‐based Sleep Champions. Survey data from 210 patients revealed a statistically significant decrease in patient‐reported noise disturbances for patients in shared rooms following the 4‐week intervention period of the HUSH program.

**Conclusion:**

The HUSH Project demonstrated that a novel multimodal intervention may be valuable in reducing sleep disruption in hospitals. These findings also indicate the benefits of using codesign methodology to support improvement projects that seek to enhance patient experiences of care.

**Patient or Public Contribution:**

This project utilised codesign methodology, which involved significant contributions from patients and consumer representatives, from research conceptualisation into intervention design, implementation and project evaluation.

## 1 INTRODUCTION

Healthcare organizations strive to facilitate healing and recovery through care and treatment, yet various environmental factors in hospitals may have negative impacts on patient recovery and wellbeing.^1^ Patients frequently report significant challenges sleeping in the hospital setting,^2^, ^3^, ^4^ which is often attributed to extrinsic factors, such as clinical interactions, ambient noise and artificial lighting.^5^, ^6^ Sleep deprivation may lead to a range of negative physical and psychological effects, including increased stress hormones, mood instability, hypertension, impaired glucose tolerance and delirium, which, ultimately, can impair recovery and safety.^1^, ^4^, ^7^, ^8^, ^9^ One of the primary causes of sleep disturbance for patients is hospital noise.^4^

To reduce sleep disruptions for patients, a range of noise‐reducing and sleep‐promoting interventions have been implemented in hospitals, including earplugs, eye masks, noise warning systems, visual reminders, quiet times and staff training.^2^, ^10^, ^11^, ^12^ Evidence from a systematic review of interventions to reduce sleep disturbances in hospitals suggests that multifaceted sleep interventions appear to be more effective than single interventions.^13^ However, a meta‐analysis of the available literature revealed that no specific intervention demonstrated the most significant impact on noise reduction.^13^ The efficacy of earplugs remains inconclusive,^12^ with research findings indicating that earplugs may be more effective in reducing background noise rather than peak noise levels.^13^ Overall, there is a lack of appropriately designed studies to evaluate the effectiveness of noise‐reducing interventions in hospitals^13^; therefore, more rigorous research is needed about improving sleep health for patients.

A systematic review and meta‐analyses investigating the effectiveness of noise reduction interventions in hospital settings indicate that there is an opportunity to enhance the design of interventions to improve sleep in hospitals.^13^ For instance, while the majority of patients (86%) used earplugs and/or eye masks when they were provided during their hospital stay,^12^ other components of multifaceted sleep interventions have demonstrated low utilisation by patients, such as ‘Do not disturb’ signs.^13^ The findings from a systematic review identified that the majority of sleep improvement projects were not designed with significant involvement from patients;^13^ thus, additional efforts may be required to design interventions that better align with patient needs and preferences. To do this, healthcare organizations may use existing participatory design methods, such as codesign.

Codesign involves actively engaging patients, healthcare providers and other stakeholders in the design and implementation of quality improvement initiatives.^14^ By including the perspectives and input of all stakeholders in the process, codesign is associated with a range of benefits, including enhanced user engagement and empowerment, improved communication and collaboration among healthcare providers, increased value creation and development of more effective and sustainable quality improvement initiatives.^15^, ^16^ The aim of this project was to explore the effectiveness of codesign in developing an intervention that could reduce sleep disruptions within the hospital setting.

## 2 METHODS

Stakeholders, including patients, consumer representatives and healthcare staff, participated in the project from its conceptualisation through to involvement in developing and implementing strategies aimed at minimising sleep disruptions. This study involved a two‐step approach: codesigning the sleep intervention and then evaluating it.

**Figure 1:**
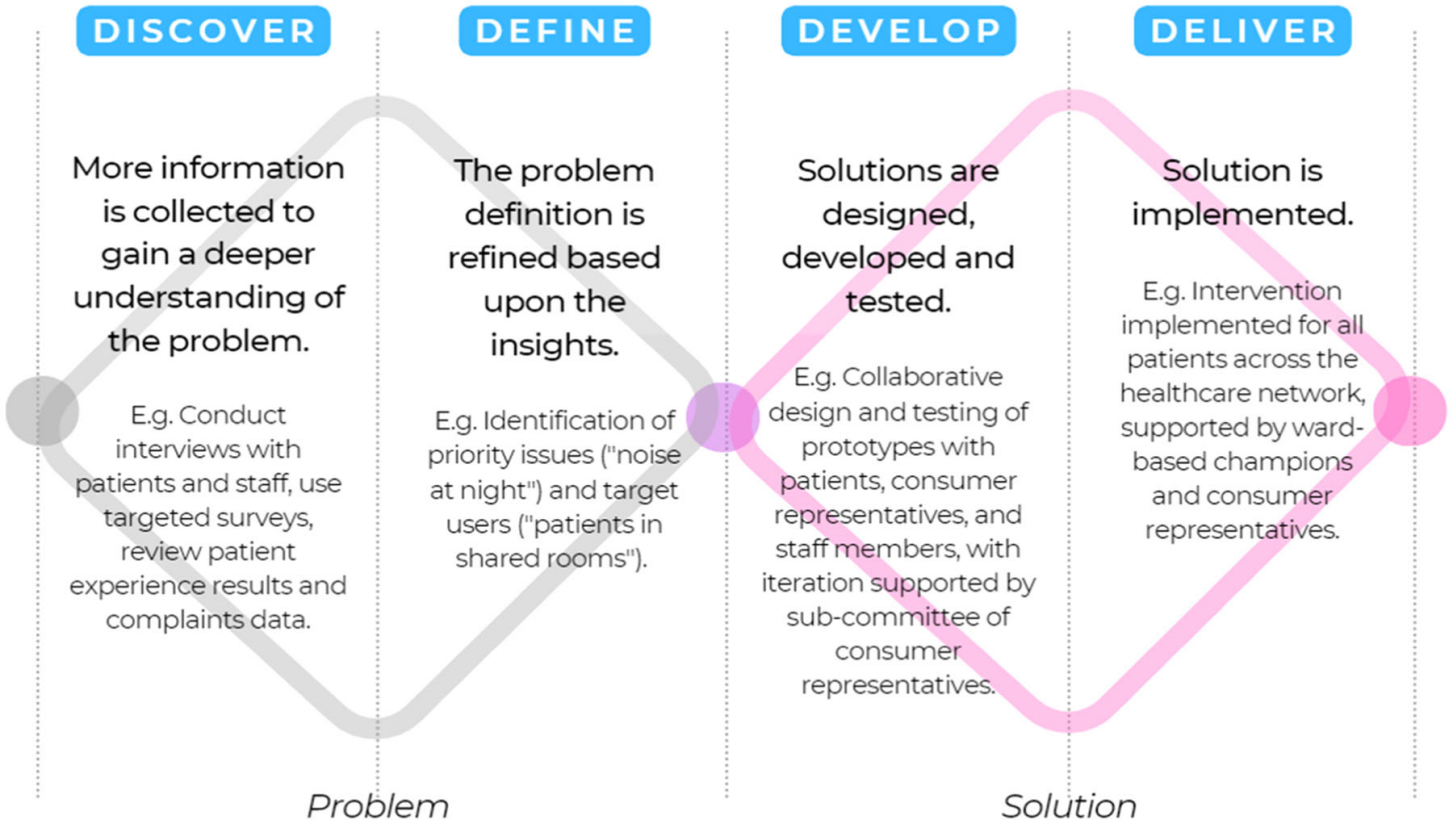
The Double Diamond model of design (adapted from Design Council, 2005).

### 2.1 Part 1: Codesign of a sleep intervention

Codesign was used to design and implement an improvement initiative that aimed to reduce sleep disruptions in the hospital setting. To guide the codesign process, the ‘Double Diamond’ framework was utilized, which employs divergent and convergent thinking in a multiphased process (see Figure 1).^17^ The Double Diamond was developed by the UK Design Council in 2005 and has been used extensively in previous research on codesign.^18^ The four phases of the Double Diamond design framework include exploring problems and gathering insights (‘Discover’), refining problems (‘Define’), creating and exploring solutions (‘Develop’) and testing and evaluating the solution (‘Deliver’).^17^

#### 2.1.1 Intervention design

To progress the improvement project, a subcommittee comprising five consumer representatives from the hospital's consumer network was established. These representatives voluntarily offered to participate and were recruited from the hospital consumer network based on their interests and experience. To facilitate their participation, fortnightly online meetings were conducted, facilitated by the lead researcher (C. A.). The consumer representatives played a critical role in the project and provided ongoing support throughout its different phases, including assessment, design, implementation and evaluation. Additionally, staff contributions were obtained through informal meetings with the Nursing Unit Managers of each ward, which allowed for flexibility and minimized disruption to clinical workflows.

#### 2.1.2 Understanding the problem

In the Discover phase, various data sources were examined, including patient experience survey data and patient complaints/grievances. In addition, new information was gathered from various stakeholders, with the researcher (C. A.) conducting interviews with both patients and staff on the wards. Next, in the Define phase, the researcher and consumer representatives carefully analysed the data and prioritized the issue of night‐time noise on the wards, which was particularly problematic in shared patient rooms.

#### 2.1.3 Developing the solution

Following this, the Develop phase focussed on generating ideas for potential solutions through a collaborative effort involving multiple stakeholders. Staff, patients and consumer representatives were all involved in this process to maximize the level of engagement and feedback garnered. To support solution ideation, the codesign process was designed to be flexible and iterative, with interviews conducted with patients and staff members on the wards. For example, staff members proposed the use of headphones for televisions at night, which was later supported by the consumer representative group and trialled with patients. The final phase (Deliver) involved implementing “the HUSH Project” across the healthcare network in all inpatient wards.

#### 2.1.4 Staff engagement

Recommendations of the Design Council highlight the importance of strong leadership in fostering a culture of innovation.^18^ Accordingly, the CEO provided executive sponsorship for the project across the healthcare network. In addition, the presence of Sleep Champions in each ward aided in the implementation. The communications and engagement plan for the project was codesigned by staff members; for example, providing herbal tea and HUSH branded pens for ward‐based clinicians. The project's successful implementation was due to the early collaboration and codesign with hospital staff, particularly nurses, and their involvement ensured that the implementation aligned with the hospital's culture and clinical processes.

### 2.2 Part 2: Evaluation

#### 2.2.1 Design

To evaluate the effectiveness of the sleep intervention, a pre‐ and post‐intervention design was used in this study.

#### 2.2.2 Sample

The inclusion criteria were adult patients (>18 years old) admitted for over 48 h to the inpatient wards at an acute tertiary hospital in Sydney, Australia. Patients with acute psychosis and/or cognitive impairment who may require additional support to safely use the interventions (i.e., earplugs and eye masks) were excluded, as well as patients admitted to short‐stay surgical wards (with length of stay less than 48 h).

#### 2.2.3 Setting

The study was conducted at a tertiary hospital in Australia, with data collected in seven inpatient wards.

*Survey materials*: Patient evaluation of sleep disruption was assessed using the publicly available ‘Sleep Disruption in Hospitals’ survey, which was developed by the Sleep Health Foundation.^19^ The survey consisted of 12 questions, with the evaluation of eight potential factors causing sleep disruption, such as noise, light, and clinical interventions. Patients rated their responses about the level of disruption to sleep for each factor using a 5‐point Likert scale (ranging from ‘not at all disruptive’ to ‘very disruptive’).

#### 2.2.4 Procedure

The study duration was 8 weeks, with 4 weeks of baseline measurement (preintervention) followed by 4 weeks of intervention and evaluation. The nursing staff provided patients with a participant information sheet about the research project. During the intervention period, patients also received a HUSH Sleep Pack on admission to the ward. After two nights in the hospital, patients were sent a text message containing a link to the survey. If patients did not have a mobile phone, nursing staff provided a paper‐based survey. Their participation was voluntary and anonymous.

#### 2.2.5 Analysis

Pre‐ and post‐intervention results were analysed using the independent samples *t* test, with data analysed using SPSS (version 12).

## 3 RESULTS

### 3.1 Part 1: Developing the intervention

**Figure 2:**
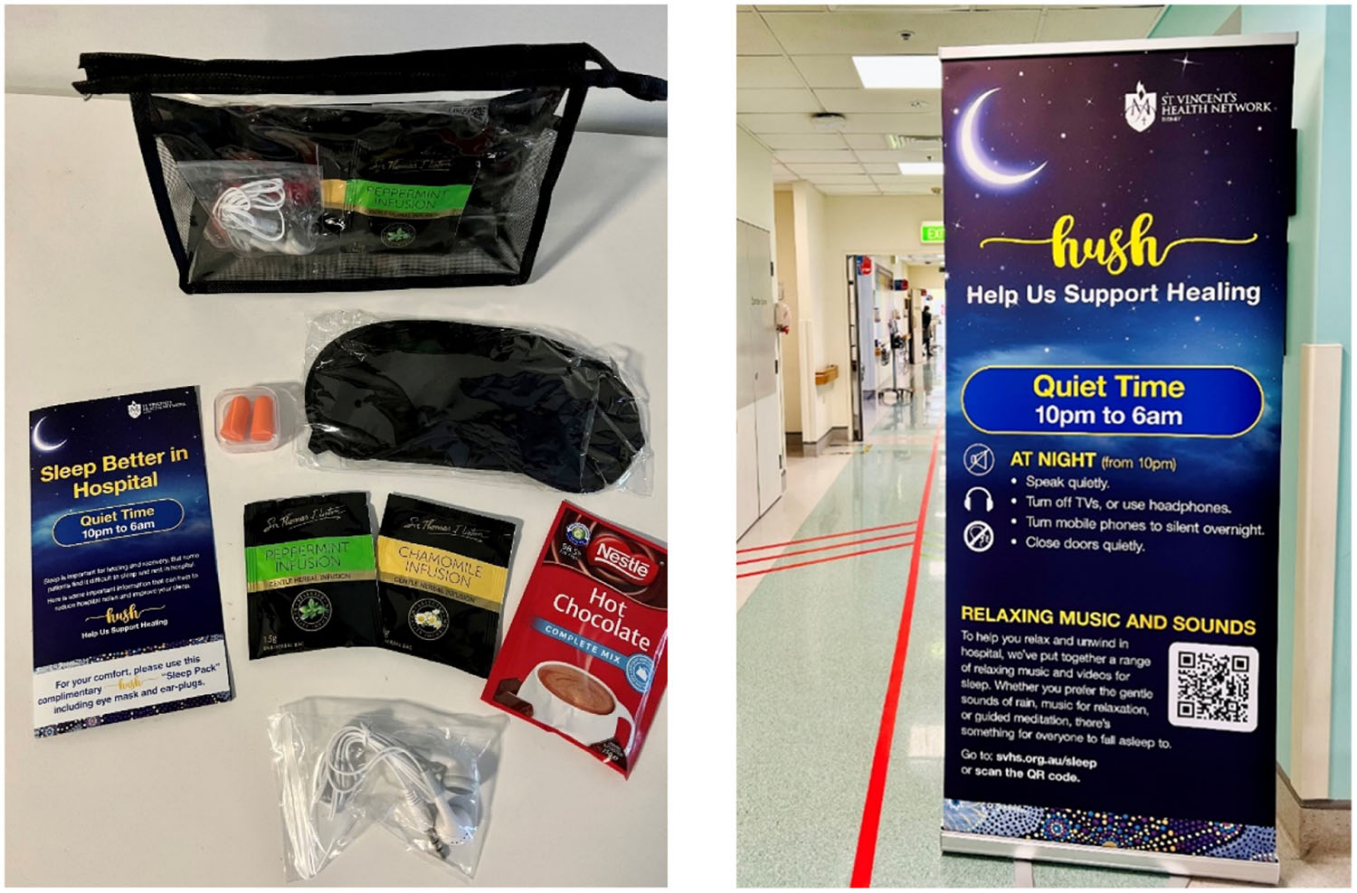
HUSH Sleep Packs and hospital signage.

The codesign methodology facilitated the development of ‘the HUSH Project’, which was created in collaboration with patients, consumer representatives and staff members to reduce sleep disruption in hospitals. This project also aligned with best‐practice recommendations for sleep in hospitals, outlined in the Sleep Health Foundation's ‘Code of Practice’.^19^ The intervention included giving patients resources to improve their sleep in the hospital, including ‘HUSH Sleep Packs’, on admission to the ward. The HUSH Sleep Packs contained earplugs, eye masks, noncaffeinated drink options (herbal tea and hot chocolate sachets) and patient information about improving sleep in the hospital (see Figure 2). Also, hospital‐wide changes were implemented, including ‘Quiet Times’ from 10:00 PM to 6:00 AM, providing 8 h of minimised noise and interruptions. Other sleep interventions included the development of a HUSH website (which was easily accessible via QR codes on the HUSH brochure) and provided access to guided meditation, white noise (rain sounds) and relaxing music.

### 3.2 Part 2: Evaluating the intervention

The Sleep Disturbances in Hospital survey was used to evaluate The HUSH intervention, with a total of 210 survey responses collected from seven inpatient wards, as shown in Table 1. Overall, 34% of respondents stayed in single rooms (*N* = 72), while 66% of respondents stayed in shared rooms (*N* = 138).

**Table 1:** Demographics of Sleep Disturbances in Hospital survey respondents.

Characteristic	*N* = 210
Sex	
Male	103 (49%)
Female	107 (51%)
Age group	
<20	3 (1.4%)
20–39	33 (15.7%)
40–59	61 (29.1%)
60–79	88 (41.9%)
80+	25 (11.9%)
Hospital room	
Single	72 (34.3%)
Shared	138 (65.7%)

Of these, the Sleep Disturbances in Hospital survey was completed by 114 patients before the HUSH intervention (i.e., no HUSH) and 96 patients following the HUSH implementation (i.e., with HUSH).

A reliability analysis was conducted on factors causing sleep disruption using the Sleep Disruption in Hospitals survey. Cronbach's alpha (*α*) showed that the Sleep Disruption in Hospitals survey reached acceptable reliability, *α* = .847.

Using the data from the Sleep Disruption in Hospitals survey, an independent two‐sample *t* test was performed to compare the sleep disruption in patients without and with the HUSH intervention, see Table 2. Overall, the results of the group comparisons were mixed. While the mean rating for some of the disturbances decreased (such as noise and acute medical conditions), these results were not statistically significant (*p* > .05).

**Table 2:** Results of Sleep Disruption in Hospitals survey.

How disruptive to your sleep in hospital was…	No HUSH (*N* = 114)		With HUSH (*N* = 96)	
	Mean	SD	Mean	SD
Noise	2.96	1.32	2.69	1.32
Light	1.91	1.04	1.94	1.14
Nursing interventions (e.g., checking vital signs)	2.21	1.13	2.29	1.12
Treatments (e.g., injections, medications)	1.96	1.05	1.93	1.11
Diagnostic tests (e.g., X‐rays, ECG)	1.42	0.79	1.35	0.77
Personal care (e.g., bathing, toileting)	1.38	0.78	1.26	0.67
Existing medical condition? (i.e., not related to your hospital stay)	1.71	1.16	1.49	0.91
Acute medical condition? (i.e., related to your hospital stay, like pain)	2.12	1.32	2.03	1.19

Abbreviation: ECG, electrocardiogram.

Following this, further analysis was conducted to compare the results for patients in shared and single rooms. For patients in shared rooms, there was a significant reduction in ratings of noise disturbances following HUSH implementation (*M* = 2.94, SD = 1.29) compared to those without HUSH (*M* = 3.4, SD = 1.27), *t*(136) = 2.07, *p* = .04. These results suggest that the HUSH intervention had a positive effect on reducing noise disruptions for patients in shared rooms.

## 4 DISCUSSION

The HUSH Project aimed to design and investigate the effectiveness of an intervention to reduce sleep disruptions in hospitals. As sleep is influenced by a range of complex factors, the HUSH Project created and applied a multimodal intervention to deliver comprehensive and holistic support for patients. Pre‐ and post‐intervention evaluations were conducted using the Sleep Disruption in Hospitals survey to measure the impact of interventions to reduce sleep disturbances in a ward setting. While overall results were mixed, the analysis indicated a statistically significant reduction in noise levels for patients in shared rooms. In this cohort, the average patient rating for noise disruption (using the Sleep Disruption in Hospital survey) improved from ‘somewhat disruptive’ to ‘a little disruptive’ within 4 weeks of program implementation. As a result of the HUSH Project, the hospital became the first healthcare facility in Australia to receive accreditation from the Sleep Health Foundation's ‘Code of Practice for Sleep Care in Hospitals’.^19^

### 4.1 Using the codesign framework in healthcare

A systematic review of sleep interventions concluded that multifaceted interventions may be effective to improve reducing sleep disruptions, yet it is essential to design sleep improvement initiatives with greater collaboration with patients and patient representatives.^13^ In response, this study utilised a codesign approach to facilitate the design process, which was guided by the Double Diamond framework. Employing divergent and convergent thinking helped mitigate risk and promptly identified potential issues, while prototyping contributed to refining the feasibility and design of interventions based on stakeholder feedback.

### 4.2 Partnering with consumers for quality improvement

Codesign is a critical method in healthcare service planning and delivery that actively involves all stakeholders to address the specific needs and concerns of patients, including consumer representatives. Although consumer representatives can provide valuable insights and perspectives, their involvement in improvement activities can be perceived as tokenistic and superficial.^20^ Codesign necessitates a partnership approach that eliminates power imbalances.^21^ Accordingly, the HUSH Project aimed to establish meaningful partnerships with consumer representatives by involving them in all aspects of project design and implementation, such as developing training materials, facilitating staff training sessions, supporting policy development and creating patient communications. The project also addressed patients' physical and emotional needs, and improved communication diversity, including creating an animated video about sleep health in hospitals for culturally and linguistically diverse patient groups and those with low health literacy.

### 4.3 Engaging clinicians in the codesign process

The Design Council recognizes the significance of engagement and relationship‐building with all stakeholders.^22^ In the healthcare setting, clinician involvement is imperative. Despite this, ineffective codesign can occur due to conflicting priorities, inadequate information sharing between service providers and users, and overlooking of staff perspectives, which may lead to reduced clinician engagement.^16^ In the HUSH Project, it was important to be accommodating to staff needs and preferences. For instance, staff collaboration was often obtained via informal meetings on the wards, which minimised disruption to clinical processes and reduced barriers to their involvement.

Staff participation ensured that the solutions met the needs of both patients and healthcare providers. For instance, staff feedback influenced the development of visually appealing pull‐up banners, specifically designed to enhance the ward environment, and the provision of disposable headphones to address noise issues from TVs in shared rooms. Also, staff feedback was necessary when setting designated times for ‘Quiet Time’ to avoid potential conflicts with clinical workflows. The HUSH Project highlighted the importance of integrating feedback from diverse stakeholders to cater to their varied needs. For example, the selection of herbal tea for the HUSH packs was altered based on pharmacist feedback about potential medication interactions. As such, effective communication and collaboration between clinical and nonclinical stakeholders was vital to ensure interventions were both person‐centred and medically appropriate.

## 5 LIMITATIONS

The limitations of this study include its limited scope, as it was conducted in a single hospital (in Sydney, Australia), and the survey was only available in English. Additionally, the evaluation was conducted immediately after project implementation, which may not provide a comprehensive understanding of the effectiveness of the sleep intervention over time. The factors influencing sleep are multifaceted and may take time to be fully implemented in an organization. Additionally, sleep improvement also involves a change in culture, which takes time to be fully integrated into the healthcare setting. Also, there is scope for a more extensive partnership with patients and stakeholders, including more diversity with consumer representation and involvement.

## 6 FUTURE RESEARCH

To further increase the generalizability of the findings, studies could be conducted in different hospital settings, such as private hospitals with single rooms, which would provide additional insights into how the interventions may be best adapted and implemented in different healthcare environments. Future research may also involve a longer evaluation period, such as 6–12 months, to gain a more comprehensive understanding of the effectiveness of the sleep intervention over time. Also, a larger sample size may permit further analysis, such as the influence of variables such as age, sex and length of stay on patient‐reported sleep measures. Additionally, further research could be conducted to identify which components of the HUSH interventions were most effective in reducing sleep disruptions for patients in hospitals.

## 7 CONCLUSION

In conclusion, the HUSH Project demonstrated the value of codesign in healthcare service planning and delivery to create an intervention that tackled the issues of importance to the project stakeholders. The results of the HUSH Project indicated a statistically significant reduction in noise disturbances for patients in shared rooms. These findings highlight the complex nature of sleep in hospitals and the need for further research to identify effective ways to mitigate sleep disruptions in hospitals. Overall, codesign is a promising approach for improving healthcare services, and healthcare organizations should consider its implementation to enhance patient experience and service quality.

## AUTHOR CONTRIBUTIONS

Corey Adams devised the project. Ramesh Walpola, Anthony Schembri and Reema Harrison were involved in the planning and supervision. Corey Adams performed the data collection and analysis, supported by Ramesh Walpola. Corey Adams wrote the article, in consultation with Ramesh Walpola, Anthony Schembri and Reema Harrison. All authors provided critical feedback and helped shape the research, analysis and manuscript.

## CONFLICT OF INTEREST STATEMENT

The authors declare no conflict of interest.

## ETHICS STATEMENT

This study was approved by the hospital Human Research Ethics Committee (HREC) in Sydney, Australia (2021/ETH11585).

## Data Availability

The data that support the findings of this study are available from the corresponding author, Corey Adams, upon reasonable request.
